# The Value of Mental Tension

**Published:** 1882-02

**Authors:** 


### THE VALUE OF MENTAL TENSION.

Dr. J. Mortimer Granville says, in *Popular Science Monthly*, for November: A certain degree of tension is indispensable to the easy and healthful discharge of mental functions. Like the national instrument of Scotland, the mind drones wofully and will discourse most dolorous music unless an expansive and resilient force within supplies the basis of quickly responsive action. No good, great, or enduring work can be safely accomplished by brain force without a reserve of strength sufficient to give buoyancy to the exercise, and, if I may so say, rhythm to the operations of the mind. Working at high pressure may be bad, but working at low pressure is incomparably worse. As a matter of experience, a sense of weariness commonly precedes collapse from "overwork;" not mere bodily or nervous fatigue, but a more or less conscious distaste for the business in hand, or perhaps for some other subject of thought or anxiety which obtrudes itself. It is the offensive or irritating burden that breaks the back. Thoroughly agreeable employment, however engrossing, stimulates the recuperative faculty while it taxes the strength, and the supply of nerve force seldom falls short of the demand. When a feeling of disgust or weariness is not experienced, this may be because the compelling sense of duty has crushed self out of thought. Nevertheless, if the will is not pleasurably excited, if it rules like a martinet, without affection or interest, there is no *verve*, and, like a complex piece of machinery working with friction and heated bearings, the mind wears itself away and a break-down ensues.

